# Significance of Phosphorylated Epidermal Growth Factor Receptor and Its Signal Transducers in Human Soft Tissue Sarcoma

**DOI:** 10.3390/ijms18061159

**Published:** 2017-05-30

**Authors:** Jia-Lin Yang, Romi Das Gupta, David Goldstein, Philip J. Crowe

**Affiliations:** 1 Department of Surgery, Clinical School of Prince of Wales Hospital, Faculty of Medicine, University of New South Wales, Sydney 2001, Australia; p.crowe@unsw.edu.au; 2 Sarcoma and Nanooncology Group, Adult Cancer Program, Lowy Cancer Research Centre, Clinical School of Prince of Wales Hospital, Faculty of Medicine, University of New South Wales, Sydney 2001, Australia; d.goldstein@unsw.edu.au; 3 Department of Paediatric and Burns Surgery, Lady Cilento Children’s Hospital, Children’s Health Queensland, Brisbane 4000, Australia; Romi_Das_Gupta@health.qld.gov.au; 4 Department of Medical Oncology, Clinical School of Prince of Wales Hospital, Faculty of Medicine, University of New South Wales, Sydney 2001, Australia

**Keywords:** EGFR, pEGFR, pAkt, pERK, soft tissue sarcoma, grade, stage, overall survival, cancer specific survival, immunohistochemistry

## Abstract

Previous studies have shown that total epidermal growth factor receptor (EGFR) protein is highly expressed in soft tissue sarcoma (STS). We aimed to investigate the significance of phosphorylated-EGFR (pEGFR) and its activated-downstream signal transducers in STS tissue samples. A tissue microarray comprising 87 STS samples was assessed for total EGFR, pEGFR and its phosphorylated signal transducers and expression was correlated with clinicopathlogical parameters including patient outcome. Although the expression of total EGFR was significantly associated with adverse STS histologic grade (*p* = 0.004) and clinical stage (*p* = 0.012) similar to pEGFR, phosphorylated protein kinase B (pAkt) and phosphorylated extracellular signal regulated kinase (pERK), it is not a prognostic factor for survival. By contrast, the expression of pEGFR is an independent factor for cancer specific survival, while pERK is an independent prognostic factor for both overall and cancer specific survival in STS (*p* < 0.05, Cox proportional hazard model and log-rank test) in addition to the recognised factors of tumour grade and clinical stage. pERK and pEGFR are new independent prognostic factors for overall and/or cancer specific survival in STS. The expression of EGFR/pEGFR, and their associated downstream signal transducers, was associated with STS progression, suggesting that EGFR downstream signalling pathways may jointly support STS cell survival.

## 1. Introduction

Sarcomas are cancers of connective tissue including hard tissue (bone and cartilage) and soft tissue (fat, fibrous tissue, muscle, blood vessel, nerve and others) with multiple forms, multiple locations, complex to treat, high mortality and social burden. Soft tissue sarcoma (STS), accounting for 70% of sarcoma, is currently treated by surgery in combination with adjuvant radiotherapy and chemotherapy. Local recurrence or metastatic disease is associated with high histologic grade and approximately 50% of these patients will eventually die of their disease [1,2]. Systemic treatment options in metastatic STS have limited efficacy and new treatments and clinically useful predictive markers are urgently required.

The expression of epidermal growth factor receptor (EGFR) protein in metastatic sarcoma cell lines was first reported in 1998 [3]. We have previously reported that in a cohort of 46 consecutive STS patients, 78% demonstrated positive expression of total EGFR [4]. This finding is consistent with other series in STS with a mean of 68% (range 60–77%) [5,6,7,8,9]. In a large Japanese study, total EGFR expression was significantly associated with histologic grade, but was not an independent prognostic factor of survival [5]. We therefore hypothesized that phosphorylated-EGFR (pEGFR) and/or its activated downstream signal transducers may be more predictive of patient outcome.

EGFR, a transmembrane tyrosine kinase receptor, is activated following its ligand binding. This leads to autophosphorylation of critical tyrosine residues which activates signalling cascades and affects gene transcription [10]. These include the Ras-Raf-MAPK (mitogen activated protein kinase), the PI3K (phosphatidylinositol 3-kinase)-Akt (Protein kinase B)-mTOR (mammalian target of rapamycin), and the JAK (Janus kinase)-STAT (signal transducers and activators of transcription) pathways. The Ras activation initiates activation of MAPK subpathways, such as extracellular signal-regulated kinase (ERK), and p38 MAPK pathways, which together with the PI3K/Akt/mTOR pathway regulate cellular proliferation, growth, survival and mobility [11]. The STATs exert diverse actions on gene transcription and protein translation, in particular STAT3 may act as an oncogene [12,13].

Use of single agent EGFR inhibitors is now a part of standard care in biologically appropriate subsets in cancers of the lung [14], colon [15], pancreas and the head and neck [10]. Those who respond may have prolonged benefit but treatments typically do not result in a cure. In STS, we and others have provided evidence that the EGFR pathway may be a potential therapeutic target [3,4,5,6,7,8,9,10]. However, there have been very few, if any, informative clinical trials or objective radiologic responses to EGFR inhibition [16]. A single arm phase II trial of single agent gefitinib (an EGFR inhibitor) in synovial sarcoma (SS) has shown a low response rate of 21% and 6% at four and six months, respectively [17], and the use of the anti-EGFR antibody Cetuximab in advanced soft tissue and bone sarcomas has also only demonstrated rates of 4.8% and 20% at four months [18], and, accordingly, the actual efficacy of EGFR inhibition in sarcoma remains unclear. Therefore, investigation of the activity of EGFR through pEGFR and its downstream signalling in sarcoma is needed for identification of predictive markers of patient survival and formulating future design of targeted therapy.

The aims of this study were to: (1) determine the expression of pEGFR and its phosphorylated signal transducers, pERK, pAkt, and phosphorylated STAT3 (pSTAT3) in primary tumour tissue samples from a cohort of patients with STS; and (2) correlate the expression of these protein markers with patients’ clinicopathological parameters and outcome.

The pERK and pEGFR were found to be independent prognostic factors for overall and/or cancer specific survival in STS.

## 2. Results

### 2.1. Clinicopathological Characteristics and Difference between Groups with or without Neoadjuvant Therapy

At the time of analysis, this cohort of 87 patients had a median follow up of 55 months (95% confidence interval 42–68 months). The clinical and pathological features of the patients are shown in Table 1. Thirteen (~15%) of the patients had neoadjuvant (preoperative adjuvant) treatment with median follow up of 40 months (95% CI: 19–61 months). The difference between the groups with (*n* = 13) and without neoadjuvant treatment (*n* = 74) is also shown in Table 1. Both groups shared many characteristics except that the neoadjuvant treatment group had significantly smaller tumour size and higher frequency of extremity origin (61%). This is because tumours in the extremities, although often smaller, usually require adjuvant therapy, as surgical margins are often on vital structures. In contrast, tumours in the retroperitoneum, particularly liposarcomas, which are often very large, are not often treated with radiotherapy due to treatment toxicity and poor evidence of benefit.

### 2.2. Correlation between Epidermal Growth Factor Receptor and Its Signalling Transducers and Clinical Stage and Tumour Grade

**Figure 1:**
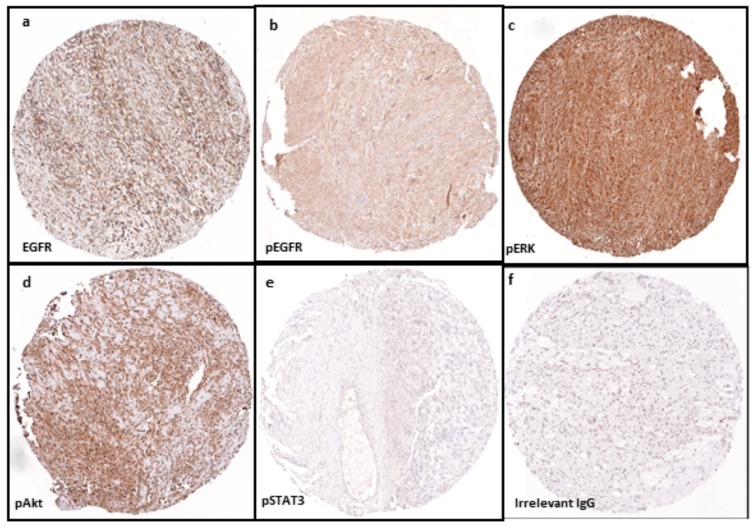
Expression of protein markers using tissue microarray technology and immunohistochemistry in a sample of leiomyosarcoma (LMS) cores (amplification ×100): (**a**) positive expression (brown colour) of total EGFR; (**b**) phosphorylated EGFR; (**c**) pERK; and (**d**) pAkt in tissue array of LMS specimen. In contrast, negative immunohistostaining (blue colour) is shown in detection of: (**e**) pSTAT3 in LMS; and (**f**) control sarcoma sample (irrelevant IgG). Abbreviations: EGFR, epidermal growth factor receptor; pEGFR, phosphorylated EGFR; pERK, phosphorylated extracellular signal-regulated kinase; p-Akt, phosphorylated protein kinase B; pSTAT3, phosphorylated signal transducers and activators of transcription-3.

Protein expression of total EGFR and pEGFR as well as their representative signalling transducers in primary tumour tissue samples of 87 patients with STS were detected by tissue microarray technology and immunohistochemistry (Figure 1). Score 0 represents negative, while positive includes 1 and 2 based on different staining intensity.

Using non-parametric tests, we identified correlation between activation of some of the biomarkers investigated and higher tumour grade and clinical stage in the entire cohort of 87 patients (Table 2), as well as in the 74 patients who did not receive neoadjuvant therapy (Table S1).

EGFR, pEGFR, pAkt and pERK were significantly intercorrelated and also significantly associated with higher histologic grade and clinical stage. In contrast, pSTAT3 expression did not correlate with EGFR, its associated other markers, or any of the clinical pathological features analysed (Table 2). 

### 2.3. Independent Prognostic Factors

Multivariate Cox proportional hazard ratio model using “forward likelihood ratio method” followed by log rank test was used to analyse all clinicopathological and biological factors and identify independent factors for overall and cancer specific survival with statistical significance (Table 3).

**Figure 2:**
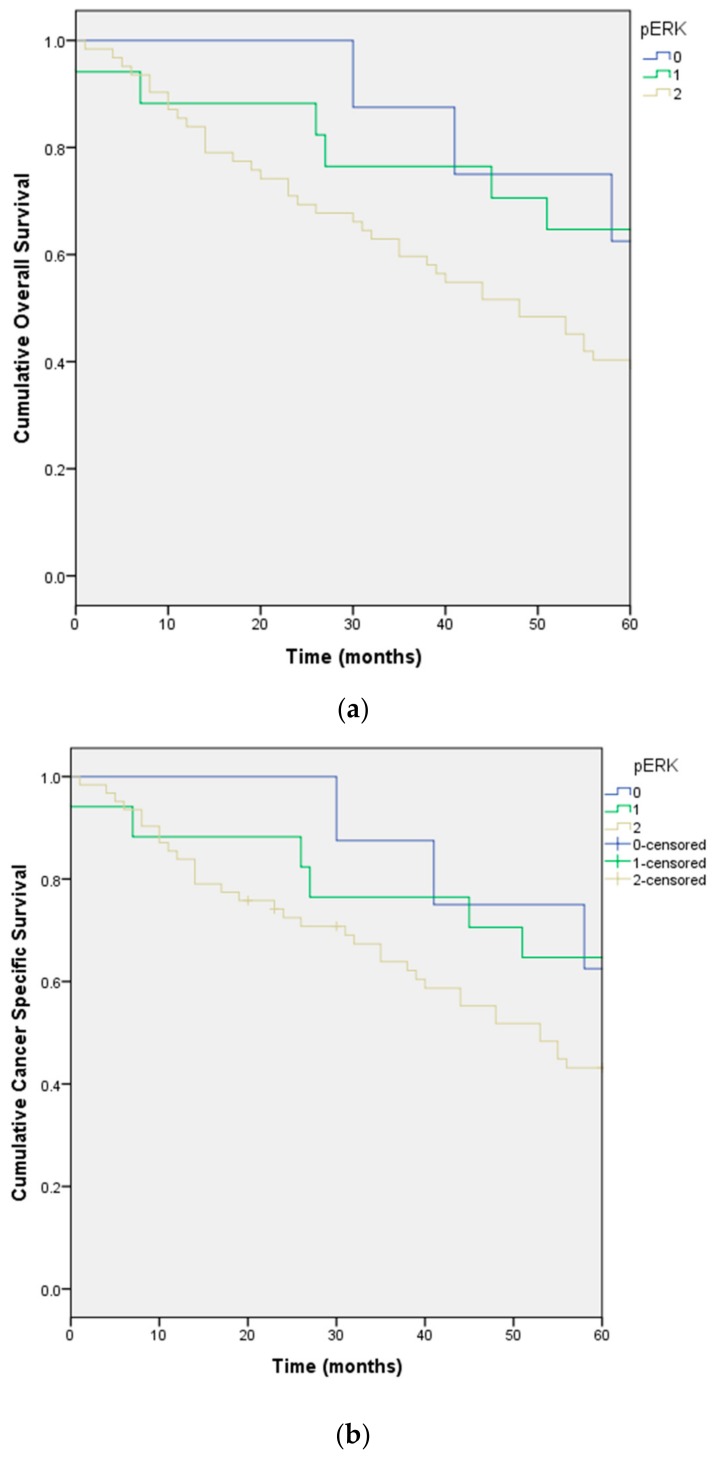
Overall survival (**a**); and cancer specific survival (**b**) stratified by differential expression of pERK in the cohort of 87 patients with soft tissue sarcoma.

Independent factors for reduced overall survival included pERK (Figure 2a,b), histologic grade and clinical stage.

**Figure 3:**
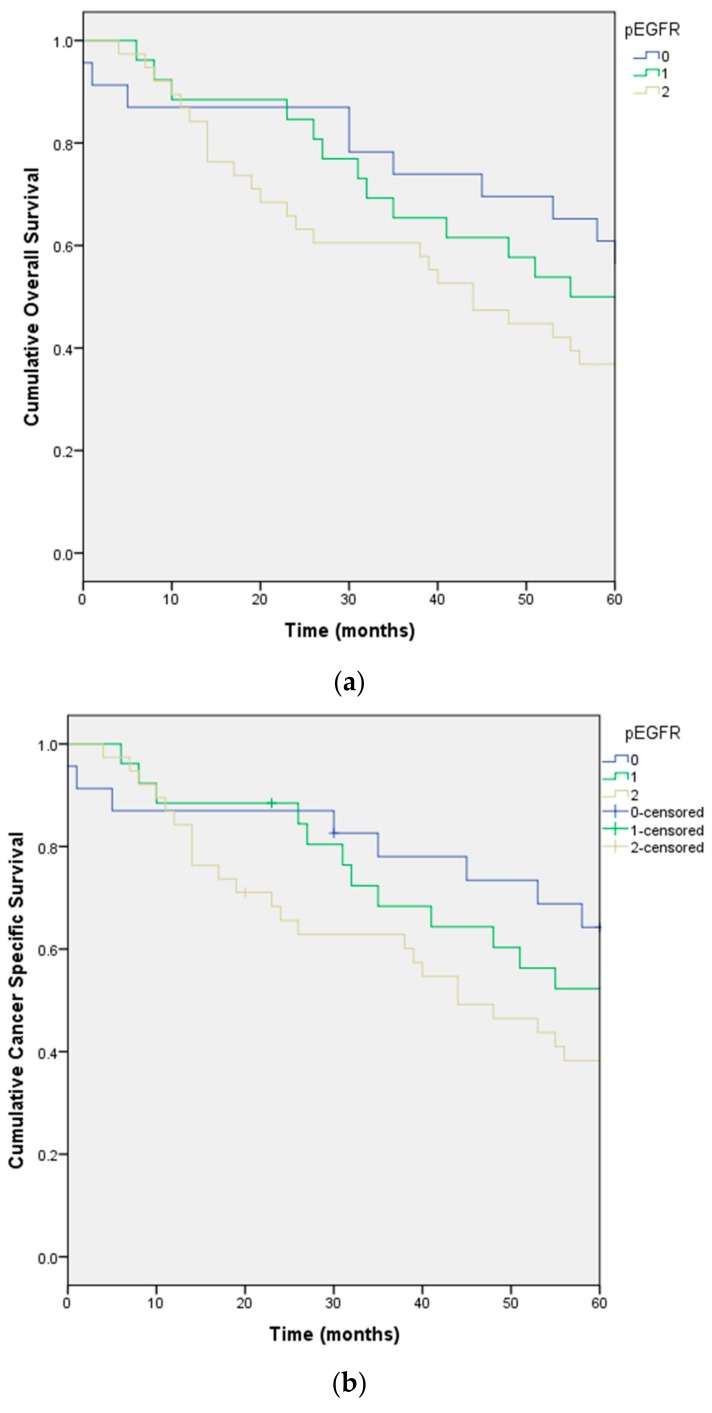
Overall survival (**a**); and cancer specific survival (**b**) stratified by differential expression of pEGFR in the cohort of 87 patients with soft tissue sarcoma.

The independent factors for cancer specific survival are also the same three factors plus pEGFR (Figure 3a,b).

## 3. Discussion

Despite multimodality treatment, Sarcoma survival rates remain unsatisfactory. Therefore, identification of new biomarkers of prognostic significance would improve current knowledge on sarcoma progression and impact on the choice of tumour therapy. There has been no report on the significance of activation (phosphorylation) of biomarkers in STS. Our results suggest an important role for activation of the EGFR pathway in sarcoma biology that may be independent of histology. We have shown for the first time that the degree of expression of pERK and pEGFR were independently and negatively associated with overall and/or cancer specific survival in our cohort of STS patients. The overexpression of pEGFR and its phosphorylated signal transducers, pAkt and pERK, apart from total EGFR, were positively associated with increased histologic grade and clinical stage. These results are new in STS, although total EGFR correlation with histologic grade has also previously been reported [5,19]. A report [20] from an ovarian cancer tissue array study on 232 cases demonstrated that pAkt expression was associated with clinical stage, also supporting our discoveries.

The independent link of pEGFR and pERK with reduced cancer specific survival in our cohort of STS patients suggests that these biomarkers may be a prognostic factor which may provide an additional parameter for clinical determination on whether to provide adjuvant therapy to appropriate STS patients, who are overexpressing this active biomarker.

Soft tissue sarcomas are a rare, heterogeneous group of mesenchymal tumours with over 70 histologic subtypes. Current treatment includes surgery and adjuvant chemotherapy and radiation therapy. Identifying an additional single therapeutic strategy for this group of cancers is dependent upon discovering a therapeutic target independent of histology. The activation of the EGFR pathway in our study was shown to occur across different STS histologic subtypes. Our results may identify a subset of STS with a shared therapeutic target [21] as well as enhancing molecular classification of the disease.

In relation to considering this pathway as a target for further exploration, we previously reported the results on detection of gene mutation on *EGFR*, *KRAS* and *BRAF* in STS cell lines [22]. Seven STS cell lines were screened for mutations in the TK domain (exons 18–24) of the *EGFR* gene and no rare sequence variants were detected. Our result is consistent with clinical reports. In a cohort of 958 patients, only two of 38 samples from the sarcoma subset were positive for any *EGFR* mutation [23]. In a synovial sarcoma study, only two of 13 tissue samples were positive for *EGFR TK* mutation, with no *EGFR* amplification on FISH analysis [24] and a further study on *EGFR* gene amplification from patients with endometrial stromal sarcoma also showed 10/10 negative results [25]. Additionally, gene mutations activating the EGFR downstream signalling pathways may mediate the primary and required resistance to EGFR-targeted therapy. It has been reported that *KRAS* and *BRAF* mutations were negatively correlated with the response to targeting EGFR treatment in lung and colorectal cancers [26,27]. On mutation analysis of *KRAS* and *BRAF* genes, all STS cell lines were found to be *KRAS* wild-type at codons 12, 13 and 61. SW872, SW982 and GCT (3/7 STS cell lines) demonstrated the *BRAF V600E* mutation (dbSNP: rs113488022, p.Val600Glu). This was consistent with previous studies [28], which showed only two of 54 samples from patients with STS had *KRAS* mutations. Similarly, although unexpectedly, we discovered that 3 STS cell lines SW872, SW982 and GCT contained a *BRAF V600E* mutation, a recent study found that none of the samples from 108 sarcoma patients were *BRAF* mutation positive [29].

In contrast, there was no correlation between expression of pSTAT3 in the cohort of sarcoma tissue samples and any clinicopathological and biological parameters detected or patient survival. This finding is consistent with our previously reported study [22]. In that study, we examined total- and phosphorylated-EGFR, ERK, Akt and STAT3 by Western blot in a panel of seven STS cell lines. We found pSTAT3 level was generally low and not increased by addition of EGF. However, pERK and pAkt levels were significantly increased by EGF stimulation. This explains the lack of correlation between pSTAT3 and other biological parameters tested. The pSTAT3 expression was not associated with pEGFR in the sample series, suggesting that pSTAT3 may not be directly linked with activation of this pathway in sarcoma. This is also consistent with a recent study in oral tongue carcinoma, in which the authors ruled out any significant association of pSTAT3 expression with tumour stage, grade, lymph node metastasis, recurrence rate, or survival [30].

Based on the present study, any subsequent trials of inhibition of EGFR and/or ERK should be restricted to this selective cohort of patients who are positive for pEGFR and/or pERK. Future studies should identify valid inhibitory and targeting drugs specifically directed at the active biomarker, pERK.

## 4. Materials and Methods

### 4.1. Patients and Samples

Archival tissue samples of primary tumour were obtained from 87 patients (Table 1) with previously untreated STS surgically resected from 1999 to 2005 at the Prince of Wales Hospital (POWH), Sydney. A prospective database has been maintained. Histological diagnoses were determined independently by two pathologists according to the classification of Einzinger and Weiss [31], as well as the criteria based on both the FNCLCC [32] and the NIH Consensus Conference systems [33]. All human tissues were obtained with consent, and experiments were carried out with the approval by South Eastern Sydney Area Health Service Research Ethics Committee on 19 December 2000 (REES 00/253).

### 4.2. Patient Treatment

After pathological confirmation of the diagnosis of soft tissue sarcoma and appropriate imaging to stage the tumour, patients were reviewed in the Soft Tissue Tumour multidisciplinary meeting POWH and a treatment plan devised. Patients with small (<5 cm) and subcutaneous tumours were treated by wide local excision only, while patients with larger and deeper tumours, below the deep fascia, were treated by wide excision and radiotherapy. A small number (*n* = 13, Table 1), generally with larger tumours were treated with pre-operative radiotherapy, using an Eibler neoadjuvant chemoradiotherapy protocol [34]. This consisted of a single radio sensitizing dose of doxorubicin followed by 30 Gy delivered in daily divided dose for 2 weeks, followed by definitive surgery 6 weeks after completion of radiotherapy. This regimen has been shown to have a similar rate of local control as standard adjuvant radiotherapy but with fewer long term side effects. Standard adjuvant radiotherapy, which consisted of 50 Gy, was delivered over 6 weeks, usually commencing 4–6 weeks after surgery. Patient were followed up clinically every 3 months for the first two years, then 6 monthly until 5 years, then annually thereafter or until lost to follow-up. All patients had a baseline MRI or CT scan 3–6 months after completing treatment, and again if any clinical abnormality was suspected and patients with high grade tumours also had a chest X-ray or a chest CT scan 6 monthly. Adjuvant post-operative chemotherapy was not offered, as there is no clear survival benefit from this treatment. Patients who developed metastatic disease were offered palliative chemotherapy.

### 4.3. Antibodies

Primary antibodies (Table S2) for immunohistochemistry were against total EGFR (Clone 31G7, Zymed Laboratories, Inc., South San Francisco, CA, USA), pEGFR (MAB3052, Chemicon International, Inc., Temecula, CA, USA), pERK (pp42/44 MAPK), pSTAT3, and pAkt (Cell Signaling Technology, Inc., Danvers, MA, USA).

### 4.4. Tissue Micro-Array (TMA) and Immunohistochemistry

A STS TMA was constructed using the ATA100™ Advanced Tissue Arrayer (CHEMICON, Temecula, CA, USA). One mm cores were taken in triplicate for each tumour from the 87 patients described above. All specimens were reviewed again and graded independently by an anatomical pathologist. Five μm sections were deparaffinized and processed using a standard immune peroxidase method. During the optimisation process, normal prostate and breast cancer tissue were used as positive controls for EGFR (Clone 31G7, Zymed Laboratories, Inc., South San Francisco, CA, USA), while colon cancer specimens were used for activated EGFR (MAB3052, Chemicon International, Inc., Temecula, CA, USA). Colon cancer was also used as the positive control for pERK and pSTAT3, while prostate cancer sections were used as positive controls for pAkt (Cell Signaling Technology, Inc., Danvers, MA, USA). Subsequently, appropriate sarcoma specimens were used as positive controls. Phosphate buffered saline (PBS) and matched irrelevant mouse or rabbit IgG1 antibodies were used as the negative controls.

The antigen retrieval method for EGFR was pepsin digestion, for pEGFR, pAkt and pERK were microwaved in citrate buffer and pSTAT3 was microwaved in EDTA. The incubation period was standardized for all antibodies to overnight at 4 °C. The concentration of primary antibodies was all 1:20 dilution except for pERK antibody that was 1:50 dilution. The Table S2 shows antigen retrieval and primary antibody details. The immunohistochemically stained slides were scanned using an Aperio ScanScope XT Slide Scanner (Aperio, CA, USA). The scoring was carried out independently by two researchers (RD and JLY), who were blinded to the clinicopathologic characteristics of the patients. A standard of semi-quantitative criteria was adapted from published methods [35,36] with modification, used for scoring the slides and was as follows: 0 (Negative): No positive staining; 1 (Weak positive): Intense staining in <10% or diffuse staining in <50% of core; 2 (Strong positive): Intense staining in ≥10% or diffuse staining in ≥50% of the core.

### 4.5. Statistical Analysis

Parametric or non-parametric statistical methods for both time-dependent and time independent variables were used where appropriate (see results). Statistical values of *p* (2-tail) < 0.05 were considered significant. Statistical analyses were performed using the IBM-SPSS Statistics 22 software (IBM-SPSS, Chicago, IL, USA).

## Figures and Tables

**Table 1:** Clinicopathological characteristics of soft tissue sarcoma patients.

Variable	All Patients (*n* = 87)		Standard Treatment ^a^ (*n* = 74)	Preoperative Adjuvant Treatment ^b^ (*n* = 13)	*p* Value ^c^ Between a and b
	Subgroup	No. (%)	No. (%)	No. (%)	
Age (years)	Mean (range)	52.5 (12–91)	52.3 (19–91)	53.6 (12–83)	0.839
	<40	23 (26.4)	20 (27.0)	3 (23.1)	0.533
	>40	64 (73.6)	54 (73.0)	10 (76.9)	
Gender	female	43 (49.4)	37 (50.0)	6 (46.2)	0.518
	male	44 (50.6)	37 (50.0)	7 (53.8)	
Histiotype ^d^	MFH/PMS	24 (27.6)	20 (27.0)	4 (30.8)	0.273
	LPS	22 (25.3)	22 (29.7)	0 (0.0)	
	LMS	16 (18.4)	12 (16.2)	4 (30.8)	
	SS	6 (6.9)	5 (6.8)	1 (7.7)	
	MPNST	4 (4.6)	3 (4.1)	1 (7.7)	
	AS	4 (4.6)	4 (5.4)	0	
	others	11 (12.6)	8 (10.8)	3 (23.1)	
Stage	I	14 (16.1)	14 (18.9)	0 (0.0)	0.377
	II	35 (40.2)	29 (39.2)	6 (46.1)	
	III	25 (28.7)	20 (27.0)	5 (38.5)	
	IV	13 (14.9)	11 (14.9)	2 (15.4)	
Grade	Low	26 (29.9)	23 (31.1)	3 (23.1)	0.319
	Intermediate	8 (9.2)	8 (10.8)	0 (0.0)	
	High	53 (60.9)	43 (58.1)	10 (76.9)	
Site	Extremity	40 (46.1)	32 (43.2)	8 (61.5)	0.034
	Trunk	14 (16.1)	12 (16.2)	2 (15.4)	
	Head and neck	7 (8.0)	7 (9.5)	0 (0.0)	
	Abdomen and pelvis	23 (26.4)	22 (29.7)	1 (7.7)	
	Thorax	3 (3.4)	1 (1.4)	2 (15.4)	
Size (cm)	<5	27 (31.0)	25 (33.8)	2 (15.4)	0.035
	>5 to <10	27 (31.0)	19 (25.6)	8 (61.5)	
	>10	33 (37.9)	30 (40.5)	3 (23.1)	
Depth	Superficial	19 (21.8)	18 (24.3)	1 (7.7)	0.166
	deep	68 (78.2)	56 (75.7)	12 (92.3)	
Margin (mm)	>10	5 (5.7)	4 (5.4)	1 (7.5)	0.995
	1–10	20 (23.0)	17 (23.0)	3 (23.1)	
	<1	12 (13.8)	10 (13.5)	2 (16.7)	
	Involved	44 (50.6)	38 (51.3)	6 (46.2)	
	Unknown	6 (6.9)	5 (6.8)	1 (7.5)	
Local Recurrence	Yes	9 (89.7)	8 (10.8)	1 (7.7)	0.597
	No	78 (10.3)	66 (89.2)	12 (92.3)	
Overall Survival (months)	Median (95% CI)	55 (42–68)	56 (45–67)	40 (19–61)	0.744

^a^ Standard treatment includes wide section only for small and/or subcutaneous tumours and surgery plus postoperative radiotherapy for high risk deep tumours; ^b^ Doxorubicin (one dose as radiosensitizer) followed by radiotherapy (28); ^c^ Statistical methods were *t* test, Chi-squared test, Fisher test or log rank test; ^d^ Histiotype indicates subtypes of soft tissue sarcomas based on histological characteristics, including MFH/PMS: malignant fibrous histiocytoma/undifferentiated pleomorphic sarcoma; LPS: liposarcoma; LMS: leiomyosarcoma; SS: synovial sarcoma; MPNST: malignant peripheral nerve sheath tumour; AS: angiosarcoma; and others.

**Table 2:** EGFR and its activated signal transducers in 87 patients with soft tissue sarcomas.

Factor		No. (%)	EGFR			pEGFR			pERK			pAkt			pSTAT3		
Score			0	1	2	0	1	2	0	1	2	0	1	2	0	1	2
**All Patients (*n* = 87)**																	
Stage	I	14 (16.1)	4 (28.6)	6 (42.8)	4 (28.6)	7 (50.0)	1 (7.1)	6 (42.9)	2 (14.3)	7 (50.0)	5 (35.7)	3 (21.4)	4 (28.6)	7 (50.0)	12 (85.7)	2 (14.3)	0 (0.0)
	II	35 (40.2)	13 (37.1)	11 (31.4)	11 (31.4)	14 (40.0)	11 (31.4)	10 (28.6)	5 (14.3)	8 (22.9)	22 (62.8)	8 (22.9)	5 (14.3)	22 (62.8)	25 (71.4)	8 (22.9)	2 (5.7)
	III	25 (28.7)	1 (4.0)	7 (28.0)	17 (68.0)	1 (4.0)	7 (28.0)	17 (68.0)	1 (4.0)	1 (4.0)	23 (92.0)	0 (0.0)	3 (12.0)	22 (88.0)	17 (68.0)	7 (28.0)	1 (4.0)
	IV	13 (14.9)	1 (7.7)	7 (53.8)	5 (38.5)	1 (7.7)	7 (53.8)	5 (38.5)	0 (0.0)	1 (7.7)	12 (92.3)	0 (0.0)	1 (7.7)	12 (92.3)	10 (76.9)	2 (15.4)	1 (7.7)
*p* Value ^a^			0.012			0.001			0.004			0.031			0.866		
Grade	L	26 (29.9)	11 (42.3)	11 (42.3)	4 (15.4)	14 (53.8)	6 (23.1)	6 (23.1)	7 (26.9)	12 (46.2)	7 (26.9)	9 (34.6)	8 (30.8)	9 (34.6)	19 (73.1)	7 (26.9)	0 (0.0)
	M	8 (9.2)	1 (12.5)	4 (50.0)	3 (37.5)	2 (57.1)	4 (28.6)	2 (14.3)	0 (0.0)	3 (28.6)	5 (71.4)	1 (14.3)	2 (28.6)	5 (57.1)	7 (85.7)	1 (14.3)	0 (0.0)
	H	53 (60.9)	7 (13.2)	16 (30.2)	30 (56.6)	7 (13.2)	16 (30.2)	30 (56.6)	1 (1.9)	2 (3.8)	50 (94.3)	1 (1.9)	3 (5.7)	49 (92.4)	38 (71.7)	11 (20.8)	4 (7.5)
*p* Value			0.004			0.001			< 0.001			< 0.001			0.484		
**Spearman’s Correlation Coefficient (*n* = 87)**																	
EGFR *R* ^b^						0.609			0.451			0.511			0.076		
*p* Value						< 0.001			< 0.001			< 0.001			0.519		
pEGFR *R*			0.609						0.536			0.671			0.095		
*p* Value			< 0.001						0.001			< 0.001			0.423		

^a^ Statistical methods used are Chi-squared test and non-parametric Kruskal–Wallis test. EGFR, epidermal growth factor receptor; pEGFR, phosphorylated epidermal growth factor receptor; pERK, phosphorylated extracellular signal-regulated kinase; pAkt, phosphorylated protein kinase B; pSTAT3, phosphorylated signal transducers and activators of transcription-3; ^b^ *R* represents correlation coefficient.

**Table 3:** Independent prognostic factors for patient survival.

Factor	Overall Survival						
	Subtype	Cox, *p*	Logrank, *p*	Median Survival (Months)	SE	95% Confidence Interval	
						Lower	Upper
pERK	0	0.006	0.008	104.0	52.4	1.3	206.6
	1			96.0	19.9	56.8	135.1
	2			48.0	7.3	33.7	62.2
Grade	Low	0.010	0.011	86.0	16.5	53.5	118.4
	mediate			55.0	2.6	49.8	60.1
	High			44.0	9.1	26.0	61.9
Stage	1	0.036	0.019	58.0	10.2	37.8	78.1
	2			81.0	6.7	67.6	94.3
	3			38.0	13.5	11.3	64.6
	4			32.0	10.7	10.8	53.1
**Factor**	**Cancer Specific Survival**						
	**Subtype**	**Cox, *p***	**Logrank, *p***	**Median Survival (Months)**	**SE**	**95% Confidence Interval**	
						**Lower**	**Upper**
pERK	0	0.013	0.016	104.0	52.3	1.3	206.6
	1			96.0	19.9	56.8	135.1
	2			55.0	6.3	42.5	67.4
pEGFR	0	0.040	0.047	86.0	10.5	65.3	106.6
	1			62.0	9.5	43.3	80.6
	2			48.0	9.6	29.1	66.8
Grade	Low	0.011	0.012	96.0	15.9	64.7	127.2
	mediate			77.0	13.6	50.2	103.7
	High			44.0	8.7	26.8	61.1
Stage	1	0.042	0.039	58.0	10.2	37.8	78.1
	2			84.0	5.3	73.4	94.5
	3			40.0	10.8	18.7	61.2
	4			32.0	10.7	10.8	53.1

## Supplementary Materials

The following are available online at www.mdpi.com/1422-0067/18/6/1159/s1.

## References

[1] Siegel R., Ma J., Zou Z., Jemal A. (2014). Cancer statistics, 2014. CA Cancer J. Clin..

[2] Kasper B., Gil T., D’Hondt V., Gebhart M., Awada A. (2007). Novel treatment strategies for soft tissue sarcoma. Crit. Rev. Oncol. Hematol..

[3] Beech D., Pollock R.E., Tsan R., Radinsky R. (1998). Epidermal growth factor receptor and insulin-like growth factor-I receptor expression and function in human soft-tissue sarcoma cells. Int. J. Oncol..

[4] Yang J.L., Hannan M.T., Russell P.J., Crowe P.J. (2006). Expression of HER1/EGFR protein in human soft tissue sarcomas. Eur. J. Surg. Oncol..

[5] Sato O., Wada T., Kawai A., Yamaguchi U., Makimoto A., Kokai Y., Yamashita T., Chuman H., Beppu Y., Tani Y., Hasegawa T. (2005). Expression of epidermal growth factor receptor, ERBB2 and Kit in adult soft tissue sarcomas. Cancer.

[6] Biscuola M., van de Vijver K., Castilla M.A., Romero-Pérez L., López-García M.Á., Díaz-Martín J., Matias-Guiu X., Oliva E., Palacios-Calvo J. (2013). Oncogene alterations in endometrial carcinosarcomas. Hum. Pathol..

[7] Teng H.W., Wang H.W., Chen W.M., Chao T.C., Hsieh Y.Y., Hsih C.H., Tzeng C.H., Chen P.C., Yen C.C. (2011). Prevalence and prognostic influence of genomic changes of EGFR pathway markers in synovial sarcoma. J. Surg. Oncol..

[8] Cascio M.J., O′Donnell R.J., Horvai A.E. (2010). Epithelioid sarcoma expresses epidermal growth factor receptor but gene amplification and kinase domain mutations are rare. Mod. Pathol..

[9] Keizman D., Issakov J., Meller I., Maimon N., Ish-Shalom M., Sher O., Merimsky O. (2009). Expression and significance of EGFR in malignant peripheral nerve sheath tumor. J. Neurooncol..

[10] Zahorowska B., Crowe P.J., Yang J.L. (2009). Combined therapies for cancer: A review of EGFR targeted monotherapy and combination treatment with other drugs. J. Cancer Res. Clin. Oncol..

[11] Morgensztern D., McLeod H.L. (2005). PI3K/Akt/mTOR pathway as a target for cancer therapy. Anticancer Drugs.

[12] Wang X., Crowe P.J., Goldstein D., Yang J.L. (2012). STAT3 inhibition, a novel approach to enhancing targeted therapy in human cancers (review). Int. J. Oncol..

[13] Quesnelle K.M., Boehm A.L., Grandis J.R. (2007). STAT-Mediated EGFR Signaling in Cancer. J. Cell Biochem..

[14] Tsujino K., Kawaguchi T., Kubo A., Aono N., Nakao K., Koh Y., Tachibana K., Isa S., Takada M., Kurata T. (2009). Response rate is associated with prolonged survival in patients with advanced non-small cell lung cancer treated with gefitinib or erlotinib. J. Thorac. Oncol..

[15] Laurent-Puig P., Cayre A., Manceau G., Buc E., Bachet J.B., Lecomte T., Rougier P., Lievre A., Landi B., Boige V., Ducreux M. (2009). Analysis of *PTEN*, *BRAF*, and *EGFR* Status in Determining Benefit From Cetuximab Therapy in Wild-Type *KRAS* Metastatic Colon Cancer. J. Clin. Oncol..

[16] Aggerholm-Pedersen N., Demuth C., Safwat A., Meldgaard P., Kassem M., Sorensen B.S. (2016). Dasatinib and Doxorubicin Treatment of Sarcoma Initiating Cells: A Possible New Treatment Strategy. Stem Cells Int..

[17] Ray-Coquard I., Le Cesne A., Whelan J.S., Schoffski P., Bui B.N., Verweij J., Marreaud S., van Glabbeke M., Hogendoorn P., Blay J.Y. (2008). A phase II study of gefitinib for patients with advanced HER-1 expressing synovial sarcoma refractory to doxorubicin-containing regimens. Oncologist.

[18] Ha H.T., Griffith K.A., Zalupski M.M., Schuetze S.M., Thomas D.G., Lucas D.R., Baker L.H., Chugh R. (2013). Phase II trial of cetuximab in patients with metastatic or locally advanced soft tissue or bone sarcoma. Am. J. Clin. Oncol..

[19] Vesely K., Jurajda M., Nenutil R., Vesela M. (2008). Expression of p53, cyclin D1 and EGFR correlates with histological grade of adult soft tissue sarcomas: A study on tissue microarrays. Neoplasma.

[20] De Graeff P., Crijns A.P., Ten Hoor K.A., Klip H.G., Hollema H., Oien K., Bartlett J.M., Wisman G.B., de Bock G.H., de Vries E.G. (2008). ERBB signalling pathway: protein expression and prognostic value in epithelial ovarian cancer. Br. J. Cancer.

[21] Lim J., Poulin N.M., Nielsen T.O. (2015). New Strategies in Sarcoma: Linking Genomic and Immunotherapy Approaches to Molecular Subtype. Clin. Cancer Res..

[22] Wang X., Goldstein D., Crowe P., Yang M., Garrett K., Zeps N., Yang J.L. (2016). Overcoming resistance of targeted EGFR monotherapy by inhibition of STAT3 escape pathway in soft tisue sarcoma. Oncotarget.

[23] Wheler J.J., Falchook G.S., Tsimberidou A.M., Hong D.S., Naing A., Piha-Paul S.A., Chen S.S., Fu S., Stephen B., Fok J.Y. (2013). Aberrations in the epidermal growth factor receptor gene in 958 patients with diverse advanced tumors: Implications for therapy. Ann. Oncol..

[24] Bode B., Frigerio S., Behnke S., Senn B., Odermatt B., Zimmermann D.R., Moch H. (2006). Mutations in the tyrosine kinase domain of the *EGFR* gene are rare in synovial sarcoma. Mod. Pathol..

[25] Capobianco G., Pili F., Contini M., de Miglio M.R., Marras V., Santeufemia D.A., Cherchi C., Dessole M., Cherchi P.L., Cossu-Rocca P. (2012). Analysis of epidermal growth factor receptor (EGFR) status in endometrial stromal sarcoma. Eur. J. Gynaec. Oncol..

[26] Benvenuti S., Sartore-Bianchi A., Di Nicolantonio F., Zanon C., Moroni M., Veronese S., Siena S., Bardelli A. (2007). Oncogenic Activation of the RAS/RAF Signaling Pathway Impairs the Response of Metastatic Colorectal Cancers to Anti-Epidermal Growth Factor Receptor Antibody Therapies. Cancer Res..

[27] Lievre A., Bachet J.B., Le Corre D., Boige V., Landi B., Emile J.F., Cote J.F., Tomasic G., Penna C., Ducreux M. (2006). KRAS mutation status is predictive of response to cetuximab therapy in colorectal cancer. Cancer Res..

[28] Kim J.I., Suh J.T., Choi K.U., Kang H.J., Shin D.H., Lee I.S., Moon T.Y., Kim W.T. (2009). Inactivation of 0^6^-methylguanine-DNA methyltransferase in soft tissue sarcomas: Association with K-ras mutations. Hum. Pathol..

[29] Je E.M., An C.H., Yoo N.J., Lee S.H. (2012). Mutational analysis of PIK3CA, JAK2, BRAF, FOXL2, IDH1, AKT1 and EZH2 oncogenes in sarcomas. APMIS.

[30] Fasanaro E., Staffieri C., Cappellesso R., Marino F., Ottaviano G., Val M., Giacomelli L., de Filippis C., Stellini E., Staffieri A. (2015). Prognostic Significance of Serine-Phosphorylated STAT3 Expression in pT1-T2 Oral Tongue Carcinoma. Clin. Exp. Otorhinolaryngol..

[31] Einzinger L., Weiss S.W. (1995). Soft Tissue Tumors.

[32] Coindre J.M., Terrier P., Guillou L., Le Doussal V., Collin F., Ranchère D., Sastre X., Vilain M.O., Bonichon F., N’Guyen Bui B. (2001). Predictive value of grade for metastasis development in the main histologic types of adult soft tissue sarcomas: A study of 1240 patients from the French Federation of Cancer Centers Sarcoma Group. Cancer.

[33] Massarweh N.N., Dickson P.V., Anaya D.A. (2015). Soft Tissue Sarcomas: Staging Principles and Prognostic Nomograms. J. Surg. Oncol..

[34] Mack L.A., Crowe P.J., Yang J.L., Schachar N.S., Morris M., Kurien E., Temple C.L.F., Lindsay R.L., Magi E., DeHaas W.G., Temple W.J. (2005). Preoperative chemoradiotherapy (modified Eilber Protocol) provides maximum local control and minimal morbidity in patients with soft tissue sarcoma. Ann. Surg. Onc..

[35] Dolled-Filhart M., Camp R.L., Kowalski D.P., Smith B.L., Rimm D.L. (2003). Tissue Microarray Analysis of Signal Transducers and Activators of Transcription 3 (Stat3) and Phospho-Stat3 (Tyr705) in Node-negative Breast Cancer Shows Nuclear Localization Is Associated with a Better Prognosis. Clin. Cancer Res..

[36] Nielsen T.O., Hsu F.D., O'Connell J.X., Gilks C.B., Sorensen P.H., Linn S., West R.B., Liu C.L., Botstein D., Brown P.O. (2003). Tissue microarray validation of epidermal growth factor receptor and SALL2 in synovial sarcoma with comparison to tumors of similar histology. Am. J. Pathol..

