# Supplementary material for: Significance of Phosphorylated Epidermal Growth Factor Receptor and Its Signal Transducers in Human Soft Tissue Sarcoma
Source: Int J Mol Sci. 2017 May 30;18(6):1159. doi: 10.3390/ijms18061159 (PMC5485983; doi:10.3390/ijms18061159)
Supplement: Supplementary file 1 [file ijms-18-01159-s001.docx]

**Table S1.** EGFR and its activated signal transducers in 74 STS patients not receiving neoadjuvant treatment.

| Factor | | No. (%) | EGFR | | | pEGFR | | | pERK | | | pAkt | | | pSTAT3 | | |
| --- | --- | --- | --- | --- | --- | --- | --- | --- | --- | --- | --- | --- | --- | --- | --- | --- | --- |
| score | |  | 0 | 1 | 2 | 0 | 1 | 2 | 0 | 1 | 2 | 0 | 1 | 2 | 0 | 1 | 2 |
| *Patients without neoadjuvant chemotherapy (n = 74)* | | | | | | | | | | | | | | | | | |
| stage | I | 14 (18.9) | 4 (26.7) | 6 (46.7) | 4 (26.7) | 7 (53.3) | 1 (33.3) | 6 (13.3) | 2 (13.3) | 7 (46.7) | 5 (40.0) | 3 (20.0) | 4 (26.7) | 7 (53.3) | 12 (86.7) | 2 (13.3) | 0 (0.0) |
|  | II | 29 (39.2) | 10 (34.5) | 10 (34.5) | 9 (31.0) | 11 (69.0) | 10 (13.8) | 8 (17.2) | 5 (17.2) | 6 (20.7) | 18 (62.1) | 8 (27.6) | 3 (10.3) | 18 (62.1) | 21 (72.4) | 7 (24.1) | 1 (3.4) |
|  | III | 20 (27.0) | 1 (5.0) | 4 (20.0) | 15 (75.0) | 1 (35.0) | 6 (10.0) | 13 (55.0) | 1 (5.0) | 0 (0.0) | 19 (95.0) | 0 (0.0) | 2 (10.0) | 18 (90.0) | 14 (70.0) | 6 (30.0) | 0 (0.0) |
|  | IV | 11 (14.9) | 1 (10.0) | 5 (40.0) | 5 (50.0) | 1 (60.0) | 6 (0.0) | 4 (40.0) | 0 (0.0) | 1 (10.0) | 10 (90.0) | 0 (0.0) | 0 (0.0) | 11 (100) | 9 (80.0) | 1 (10.0) | 1 (10.0) |
| *p* Value ^a^ | | | 0.030 | | | 0.007 | | | 0.003 | | | 0.013 | | | 0.558 | | |
| grade | L | 23 (31.1) | 10 (43.5) | 9 (39.1) | 4 (17.4) | 13 (56.5) | 4 (17.4) | 6 (26.1) | 7 (30.4) | 9 (39.1) | 7 (30.4) | 9 (39.1) | 6 (26.1) | 8 (34.8) | 17 (73.9) | 6 (26.1) | 0 (0.0) |
|  | M | 8 (10.8) | 1 (12.5) | 4 (50.0) | 3 (37.5) | 2 (25.0) | 4 (50.0) | 2 (25.0) | 0 (0.0) | 3 (37.5) | 5 (50.0) | 1 (12.5) | 2 (25.0) | 5 (50.0) | 7 (85.5) | 1 (12.5) | 0 (0.0) |
|  | H | 43 (58.1) | 5 (11.6) | 12 (27.9) | 26 (60.5) | 5 (11.6) | 15 (34.9) | 23 (53.5) | 1 (2.3) | 2 (4.6) | 40 (93.1) | 1 (2.3) | 1 (2.3) | 41 (95.4) | 32 (74.5) | 9 (20.9) | 2 (4.6) |
| *p* Value | | | 0.005 | | | 0.002 | | | < 0.001 | | | < 0.001 | | | 0.710 | | |
| *Spearman’s correlation coefficient (n = 74)* | | | | | | | | | | | | | | | | | |
| EGFR *R* ^b^ | | |  | | | 0.571 | | | 0.394 | | | 0.511 | | | 0.076 | | |
| *p* Value | | |  | | | < 0.001 | | | 0.001 | | | < 0.001 | | | 0.519 | | |
| pEGFR *R* | | | 0.571 | | |  | | | 0.429 | | | 0.508 | | | 0.095 | | |
| *p* Value | | | < 0.001 | | |  | | | < 0.001 | | | < 0.001 | | | 0.432 | | |

^a^ Statistical methods used, are Chi-squared test and non-parametric Kruskal-Wallis test. EGFR = epidermal growth factor receptor, pEGFR = phosphorylated epidermal growth factor receptor, pERK = phosphorylated extracellular signal-regulated kinase, pAkt = phosphorylated protein kinase B, pSTAT3 = phosphorylated signal transducers and activators of transcription-3. ^b^ *R* represents correlation coefficient.

**Table S2**. Antibodies selected for immunohistochemistry on tissue microarray.

**Antibody Source Dilution Antigen Retrieval Incubation 2° Antibody Visualisation**

EGFR (MM) Zymed 1:20 Pepsin digestion Overnight @4°C anti-mouse ABC Vectastain/DAB

Activated EGFR (MM) Chemicon 1:20 1 X Citrate buffer/PBS Overnight @4°C anti-mouse ABC Vectastain/DAB

pAkt (RP) Cell Signaling 1:20 1 X Citrate buffer/TBST Overnight @4°C anti-rabbit ABC Vectastain/DAB

p44/42 MAPK (RP) Cell Signaling 1:50 1 X Citrate buffer/PBS Overnight @4°C anti-rabbit ABC Vectastain/DAB
(Erk1/Erk2)

pSTAT3 (RM) Cell Signaling 1:20 1mM EDTA (pH 8) Overnight @4°C anti-rabbit ABC Vectastain/DAB

MM: Mouse monoclonal antibody, RP: Rabbit polyclonal antibody, RM: Rabbit monoclonal, PBS: Phosphate buffered saline,

TBST: 1XTris buffered saline/0.1% Tween-20, EDTA: Ethylenediaminetetraacetate disodium dehydrate, DAB: 3,3'-diaminobenzidine. Secondary antibodies, ABC Vectastain® and DAB were obtained from Vector Laboratories, Burlingame, CA.
